# Effect of an elongation bending derotation brace on the infantile or juvenile scoliosis

**DOI:** 10.1186/s13013-018-0160-4

**Published:** 2018-08-07

**Authors:** John Thometz, XueCheng Liu, Robert Rizza, Ian English, Sergery Tarima

**Affiliations:** 1 Department of Orthopedic Surgery, Children’s Hospital of Wisconsin, Medical College of Wisconsin, Milwaukee, WI USA; 2 Musculoskeletal Functional Assessment Center, Children’s Hospital of Wisconsin, Medical College of Wisconsin, Milwaukee, WI USA; 3 Department of Mechanical Engineering, Milwaukee School of Engineering, Milwaukee, WI USA; 4 Division of Biostatistics, Institution for Health & Society, Medical College of Wisconsin, Milwaukee, WI USA; 5 Pediatric Orthopaedics, 9000 W. Wisconsin Ave., Suite 360, PO Box 1997, Milwaukee, WI 53201 USA

**Keywords:** Early onset scoliosis, CAD/CAM, EBDB, Cobb angle

## Abstract

**Background:**

A wide variety of braces are commercially available designed for the adolescent idiopathic scoliosis (AIS), but very few braces for infantile scoliosis (IS) or juvenile scoliosis (JS). The goals of this study were: 1) to briefly introduce an elongation bending derotation brace (EBDB) in the treatment of IS or JS; 2) to investigate changes of Cobb angles in the AP view of X-ray between in and out of the EBDB at 0, 3, 6, 9, and 12 months; 3) to compare differences of Cobb angles (out of brace) in 3, 6, 9, and12 month with the baseline; 4) to investigate changes (out of brace) in JS and IS groups separately.

**Methods:**

Thirty-eight patients with IS or JS were recruited retrospectively for this study. Spinal manipulation was performed using a stockinet. This was done simultaneously with a surface topography scan. The procedure was done in the operating room for IS, or in a clinical setting for JS. The brace was edited and fabricated using CAD/CAM method. Radiographs were recorded in and out of bracing approximately every 3 months from baseline to 12 months. A linear mixed effects model was used to compare in and out of bracing, and out of brace Cobb angle change over the 12 month period.

**Results:**

Overall, 37.5% of curves are corrected and 37.5% stabilized after 12 months (Thoracic curves 48% correction, 19% stabilization; thoracolumbar curves 33% correction, 56% stabilization and lumbar curves 29% correction, 50% stabilization). The juvenile group had 25.7% correction and 42.9% stabilization, while the infantile group had 50% correction and 32.1% stabilization. There was a significant Cobb angle in-brace reduction in the thoracic (11°), thoracolumbar (12°), and lumbar (12°) (*p* < 0.001). There was no statistically significant change in out of brace Cobb angle from baseline to month 12 (*p* > 0.05). No patients required surgery within the 12 month span.

**Conclusions:**

This study describes a new clinical protocol in the development of the EBDB. Short-term results show brace is effective in preventing IS or JS curve progression over a 12 month span.

## Background

Approximately 70 years ago, Dr. Blount and Dr. Schmidt from our institution developed the Milwaukee brace to control curve progression for children with idiopathic scoliosis. Since then, a variety of other types of braces have been utilized clinically. The choices of bracing are prescribed based on the type of spinal deformity, and the extent of their success is due to bracing design and patient compliance [1–3]. For children with infantile scoliosis (IS) or juvenile scoliosis (JS), the Milwaukee brace has been preferred over a thoraco-lumbar-sacral orthosis (TLSO). Bracing may lead to rib cage distortion and create a reduction in pulmonary function [4–6]. The design of the Milwaukee brace makes it preferable for the upper thoracic curvature [7].

Most universal designs, including the Milwaukee and most TLSO braces, follow a symmetric pattern. These usually apply a force to the apex of the curve through foam pads integrated to the bracing design by the orthotist. However, symmetric bracing do not provide the same in-brace correction as seen in asymmetric bracing, which is a common indication of long term out of bracing success [1, 8, 9]. Additionally, not all patients will be able to tolerate a symmetric brace, since an asymmetric brace aims to provide a customized fit to improve wearability and comfort, and reduce brace weight [1, 10, 11]. One of the most popular asymmetric bracing includes the Chêneau brace, which aims to provide correction through a system of multipoint pressure zones [12, 13]. While these braces have proven to provide one of the highest in brace (IB) corrections, the indication for the use of these orthoses is to treat children with adolescent scoliosis [1]. Additionally, the Cheneau-Rigo brace includes a classification system with different types of braces, which allows the orthotists to design the brace.

Many clinicians perform serial casting on younger patients, since early-onset of scoliosis (EOS) patients are immature and have the largest potential for recovery through non-operative treatments [14]. It is common practice for bracing to be prescribed after casting to maintain the initial correction. Bracing is also prescribed to patients who are not able to tolerate casting [15]. Rather than serve as a corrective force, bracing will aim to halt curve progression, prevent respiratory dysfunctions, reduce pain, and enhance posture and cosmetic appearance [1, 15].

Overall, bracing studies are usually done on AIS populations. Although few are done on juvenile idiopathic scoliosis (JIS), or infantile idiopathic scoliosis (IIS), few studies have investigated the effects of bracing on the EOS population following a spinal manipulation procedure. To our knowledge, this will be the first study to investigate the effects of a computer aided design (CAD) and computer aided manufacturing (CAM) brace on patients with EOS. We will also introduce a new CAD/CAM bracing design, known as an elongation bending derotation brace (EBDB), which integrates the spinal manipulation procedure into its design. The purpose of this study was: 1) to briefly describe the preliminary results using the new EBDB in the treatment of IS or JS; 2) to investigate changes of Cobb angles in the AP view of X-ray between in and out of the EBDB bracing; 3) to compare differences of out of brace (OOB) Cobb angles in 3, 6, 9, and12 month with baseline; 4) to investigate OOB changes in JS and IS groups separately.

## Methods

### Study recruitment

Thirty-eight patients (22 males, 16 females; 17 IS, 21 JS) were recruited retrospectively for this study. 9 children were diagnosed with neuromuscular scoliosis, 1 congenital scoliosis, and 28 with IIS or JIS. This study was approved by IRB committee at Children’s Hospital of Wisconsin. At the time of use of the EBDB, the average age was 6.2 years old (ranging from 4 months to 10 year-old). Criteria for inclusion includes: 1) All subjects are diagnosed with IS or JS (idiopathic, neuromuscular, or congenital); 2) Subjects must have not had any type of spinal surgery prior to bracing treatment; 3) Must be under 10 years old during the time of their first scan; 4) Must have had at least one follow up visit after their baseline scan before the 12 month mark.

Before their customized bracing treatment, 25 patients received some type of treatment (13 received TLSO bracing only, 8 received a series of casts only, 3 patients received casting and TLSO bracing, 1 patient received Physical therapy). 13 other patients received no prior treatment.

### Spinal manipulation, surface topography

In a clinic setting, the physicians determined the correction needed from the patients x-rays. The patient stood still in front of the physician with their arm held above their heads by the assistant. The physician used stockinet straps to provide translational and de-rotation force to correct the scoliosis curve. This allows for manipulation of the curve in the coronal and transverse plane, while also provide longitudinal traction by holding the upper limbs. While the patient was in the corrected position, a trained assistant used the handheld scanner (Polhemus FastSCAN Scorpion, Colchester, VT) to create a 3D scan of the patient’s torso from the armpits down to the bilateral greater trochanter of the femur.

If the patient is unable to stand still in the clinic setting, the scanning was done in the OR while the patient was under general anesthesia. The patient was placed on a Spica casting table. Longitudinal traction was applied to the patient’s arms and leg by assistants. A translational and de-rotation force was applied the scoliosis curve with stockinet straps to correct the curve in the coronal and transversal plane, with a mechanism was similar to the procedures mentioned above. The process differs in that the child is positioned with a much more dramatic bending movement used. The strap is similar.

### CAD, CAM and Fitting

Using a computerized aided design, the 3D shape of EBDB was created and sent to manufacturing. Afterwards, the patient returned to the clinic for a brace fitting. During fitting, the orthotist provided any necessary adjustments to the orthosis to make sure the orthosis fits properly. The costs of orthosis for children on the spica table with or without sedation are the same as TLSO, but additional charges are billed to children who needs to receive general anesthesia in OR. However, we have to remember that the brace is being used as an alternative to the cast for infantile scoliosis. Juvenile patients done in clinic have a similar charge for the brace.

### Radiographic analysis

All children were radiographically evaluated before their bracing treatment and in their brace during the day of fitting, which serves as our IB and OOB baselines. Follow up radiographic analyses were measured at approximately 3, 6, 9, or 12 months after the baseline scan, with missing measurements interpolated between the closest visits. Curve segments are classified to thoracic (T), thoracolumbar (TL), and lumbar (L) categories.

### Statistical analysis

We determined individual success of curve treatment based on the Scoliosis Research Society (SRS) criteria of spine correction for AIS, but followed up after approximately 12 months and used the criteria for EOS [16]. A ≥ 6° change or higher in Cobb angle indicates progression, ≤ − 6°change or lower indicates correction, while a range of changes between ≤5° or lower and ≥ − 5° or higher indicates stabilization. Average Cobb angle change between IB and OOB were measured and compared after the data was standardized in terms of gender, age, bracing treatment time using a linear mixed effects model with random intercepts and fitted angles. This model was also used to test changes of Cobb angles for OOB from 0 to 12 months, and the interaction effects of age and gender on spinal curvature. Additionally, a Wilcoxon signed-rank test was applied to evaluate the effects of bracing at baseline for IS, JS, and combined groups. In a separate analysis, the IS and JS groups had their OOB Cobb angle changes compared separately. A *p* value of less than 0.05 is considered significant.

## Results

The EBDB management protocols has been used in terms of children standing or supine position. In standing or supine position, children’s spine was manipulated to correct the curvatures in three planes, then the corrected spine was scanned.

The starting Cobb angle was 38 ± 14° (std) in the thoracic (ranging from 19° to 68°), 30 ± 9.6° in the thoracolumbar (ranging from 19° to 42°), and 36 ± 10.3° in the lumbar sections (ranging from 22° to 53°). No patients required surgery within the 12 month span. The findings for OOB Cobb angle changes are shown in Table 1. There were no significant differences of curves in terms of age and gender (*p* > 0.05).

**Table 1:** Cobb angle changes in children with out of brace over time (a Linear mixed effect model, *n* = 36, *P* > 0.05)

Levels of Curve	Month	Cobb Angle (°)	Curve change (°)	% Change
Thoracic	0	38.0 ± 14.0	NA	NA
	3	30.1 ± 19.7	−5.6	−15.6%
	6	30.2 ± 21.5	−5.5	− 15.5%
	9	31.5 ± 24.2	−4.2	−11.6%
	12	29.4 ± 24.3	−6.2	−17.5%
Thoracolumbar	0	30.0 ± 9.6	NA	NA
	3	25.2 ± 11.2	0.2	0.6%
	6	24.8 ± 11.6	−0.2	−0.9%
	9	24.3 ± 10.3	−0.7	−2.7%
	12	23.9 ± 10.0	−1.1	−4.5%
Lumbar	0	36.0 ± 10.3	NA	NA
	3	25.4 ± 14.3	−3.5	−12.2%
	6	27.9 ± 14.5	−1	−3.5%
	9	30.2 ± 14.2	1.3	4.5%
	12	29.9 ± 14.2	1	3.6%

**Fig. 1:**
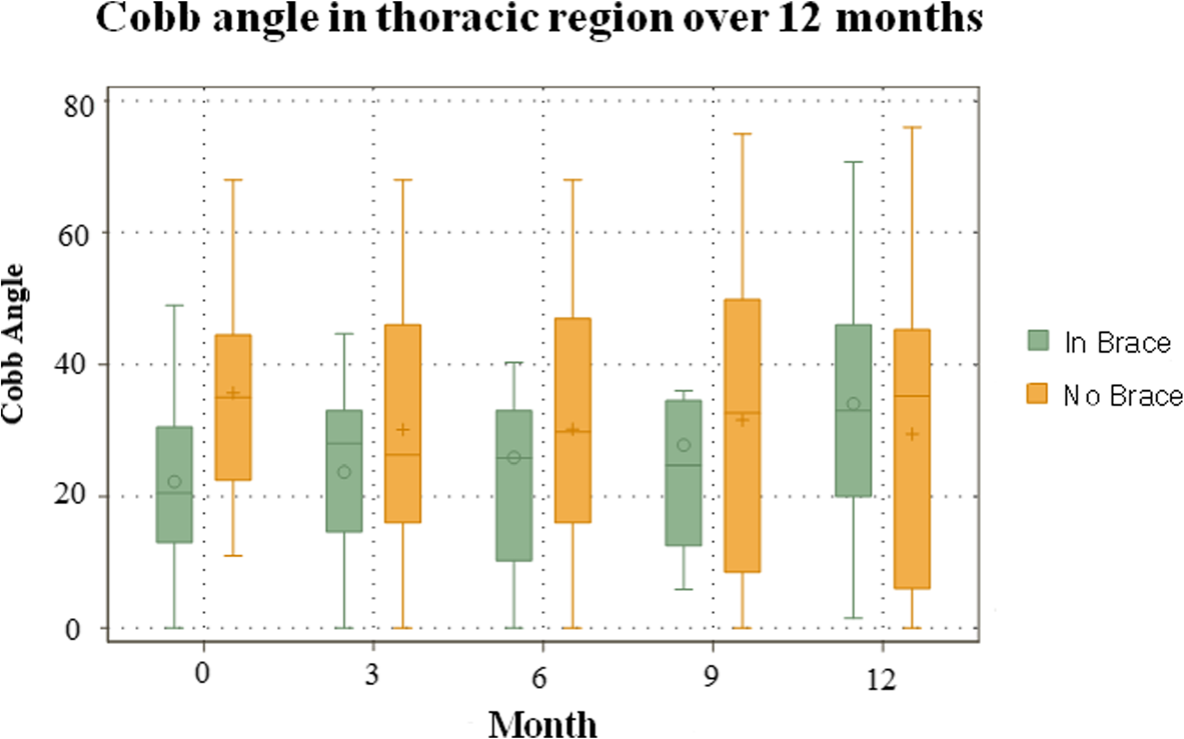
IB and OOB Cobb angle changes for the thoracic region over a 12 month span

**Fig. 2:**
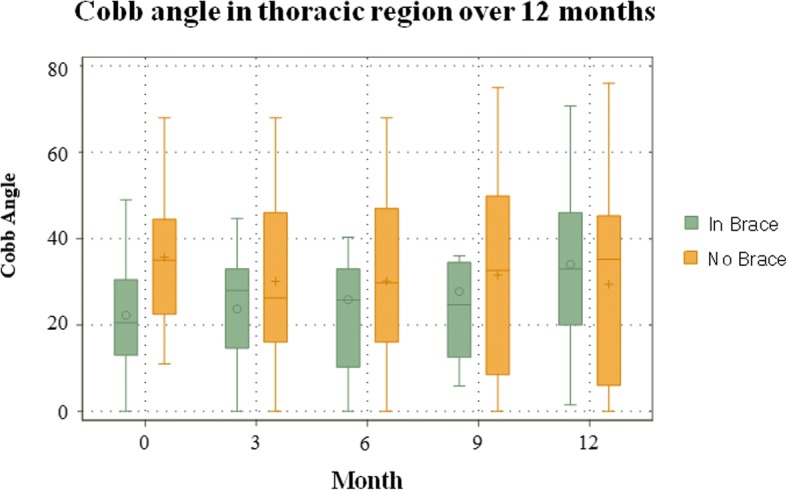
OOB Juvenile and infantile Cobb angle changes over a 12 month span

When compared to the baseline radiographic measurements, the in-brace correction reduced the Cobb angle from 38° to 24.2° in the thoracic (36.3% reduction), 30° to 10.3° in the thoracolumbar (65.7% reduction), and 36° to 18.5° in the lumbar (48.3% reduction). The juvenile group had 23% correction, 47% stabilization, and 30% progression of curves. The infantile group had 50% correction, 32% stabilization, and 18% progression of curves. After the data was standardized in terms of age, gender, and time using a linear mixed effects model, significant in-brace changes were found in the T (− 11° reduction), TL (− 12° reduction), and L segment (− 12° reduction) (*P* < 0.001). Between juvenile and infantile scoliosis, overall, there was no significant difference in Cobb angle (*P* > 0.05). Changes between OOB and IB shows significant change for the IS, JS, and combined age groups (P < 0.001). There was no significant difference in Cobb angle changes over time (*p* > 0.05). Figures 1 and 2 show the Cobb angle changes in the thoracic region, and the OOB IS and JS groups, respectively. The curve improvement in one congenital scoliosis patient was in the compensatory curves.

## Discussion

The EBDB treatment showed to be effective in correcting nearly half of the thoracic curves and one third of the other curves. When combining all data curves, 75% of curves were corrected or stabilized and 25% of curves were progressed. The efficacy of our brace is further supported by finding no statistical significance of Cobb angle changes in relation to time in all three spinal segments.

Since children with EOS have ongoing spinal growth and development, EDF casting needs to be repeated every couple of months. This may be less cost effective and less patient friendly because visits are more frequent and may require casting to be done in the OR with the patient under general anesthesia. The brace technique does not need to repeat the casting process. Additionally, CAD/CAM modification holds a significantly reduced rectification time by 108 min (63.5% reduction) [7, 17]. However, we do bill the time of manipulation for scoliotic spine.

CAD/CAM can be done in different approaches, and has been shown to be equal to or improved over traditional methods. Wong et al. found a similar efficacy between TLSO fabrication through plaster molding compared to fabrication through CAD/CAM for IB reduction. They reported 41.9% in-brace reduction using CAD/CAM (− 12.8°) and 32.1% in-brace reduction using traditional approach (− 9.8°) [18]. Others integrated CAD/CAM with a finite element analysis (FEA) to fabricate bracing [11, 17]. Desbiens-Blais et al. did this using Boston brace guidelines, and found a similar efficacy to the traditional TLSO brace. They had an IB correction of 16° using their method vs. 11° with a TLSO for thoracic curves and 13° vs 16° for Thoracolumbar/Lumbar curves (*p* > 0.05) [17]. While these studies are done on AIS patients, Sankar et al. found a better in-brace correction and increased comfort in a population of 10 scoliosis patients of various etiologies [19]. He used compared the CAD/CAM method to the traditional methods [19] Although their population is smaller and their patients’ IB radiography was recorded 3 months after baseline, they found the highest percent in-brace correction of 51% in CAD/CAM and 44% in TLSO [19]. Our baseline in-brace corrections of 37.5% thoracic, 45.6% thoracolumbar, and 51.9% lumbar indicates the EBDB has a similar efficacy as compared to most CAD/CAM bracing studies.

This is a study utilizing a novel approach to create the CAD/CAM brace. This is clinical treatment protocol that will integrate the use of manipulation of the spine in its bracing design through either standing or supine position. Manipulation mechanisms will involve in the corrective forces by pulling the stockinet. Younger patients who are unable to stand still during spinal manipulation will be required to have these procedures in the OR, while older patients are done in the clinic. Thus the EBDB provides not only a CAD/CAM based asymmetric brace, but also adequate 3D correction of the spine deformity by manipulation.

There have been rare bracing studies on the IIS population. There is a case report of a 2 year-old with scoliosis due to Marfan’s syndrome; there was a 12° IB correction and a 22° OOB reduction from baseline after approximately 2.5 years [20]. In our study, we found a comparable 11° (T), 12° (TL), and 12° (L) in-brace reduction (*P* < 0.001). Studies on younger populations usually investigate the effects of universal bracing designs on JIS populations. Their findings often have a wide variation due to differences in follow up timing, compliance, prescribed daily bracing wear, classification of curve change results, and study population [2, 4, 21–25]. Aulisa et al’s prospective bracing study found that after a 24 month follow up using a Milwaukee, Lyon, or PSAB brace, OOB Cobb angle decreased from 29.6° to 16.9° (12.7° difference), and 77.8% of patients had spinal correction while 15.9% obtained stabilization [4]. However, another study only found an OOB change of 4° after 4 years using an Edinburgh brace, which is longer in duration compared to most bracing studies [21]. Tolo et al’s study on JIS treatments found a 13% OOB Cobb angle reduction after 3 years of Milwaukee bracing treatment [22], but our results were only reported in one year follow-up and they were not comparable with studies with longer term follow-up. Overall, 48.3–56% of patients with naturally progressed JIS needed operation by the time they reach skeletal maturity, and 70% of all JIS curves progress over time [21, 22, 25, 26].

### Limitations

While our study has radiographic patient measurements every 3 months, missing measurements are interpolated using a linear regression analysis. Similar to most bracing studies, compliance can always be an issue in bracing studies. Our results with one year follow-up are preliminary and were not comparable with studies with longer follow-up. We would also like to extend the duration of our study to two years to validate the long term effect of the EBDB.

## Conclusions

The early onset of scoliosis can be treated with a custom fitted, asymmetric brace (EBDB) that integrates the 3D spinal manipulation correction into its design. It may provide users with a more patient friendly approach to treating EOS. This is especially helpful for patients who are not able to tolerate universal symmetric TLSO designs or repeated casting treatments.

## Abbreviations

CAD: Computer aided design
CAM: Computer aided manufacturing
EBDB: An elongation bending derotation brace (also previously named as Milwaukee Contoured Corrective CAD/CAM based brace, Mi3C™)
EDF: Elongation-derotation-flexion
EOS: Early onset of scoliosis
FEA: Finite element analysis
IB: In brace
IIS: Infantile idiopathic scoliosis
IS: Infantile scoliosis
JIS: Juvenile idiopathic scoliosis
JS: Juvenile scoliosis
L: Lumbar
OOB: Out of brace
Std: Standard deviation
T: Thoracic
TL: Thoracolumbar
TLSO: Thoraco-lumbar-sacral orthosis
